# Role of serotonin in modulation of decision-making in Parkinson’s disease

**DOI:** 10.1177/02698811221144636

**Published:** 2023-01-11

**Authors:** Lisa Nobis, Maria Raquel Maio, Youssuf Saleh, Sanjay Manohar, Annika Kienast, Emily McGann, Masud Husain

**Affiliations:** 1 Oxford Centre for Human Brain Activity, Wellcome Centre for Integrative Neuroimaging, Department of Psychiatry, University of Oxford, Oxford, UK; 2 Nuffield Department of Clinical Neurosciences, University of Oxford, Oxford, UK; 3 Department of Experimental Psychology, University of Oxford, Oxford, UK

**Keywords:** Decision-making, Parkinson’s disease, serotonin

## Abstract

**Background:**

Dysfunction of dopaminergic pathways has been considered to play a pivotal role in Parkinson’s disease (PD), affecting the processing of emotional and rewarding information, and potentially leading to symptoms of depression or apathy. However, some aspects of motivation in PD might be affected by non-dopaminergic mechanisms.

**Aim and method:**

The objective of this experimental medicine study was to investigate the contribution of serotonergic modulation via administration of citalopram (20 mg) for 7 days on motivated decision-making in twenty PD patients, measured using several different computerised tasks and clinical questionnaires that probe different aspects of decision-making. Twenty healthy controls were additionally tested without medication to assess any baseline differences between the two groups.

**Results:**

Results indicated that PD patients were overall less motivated than controls on an effort- and reward-based decision-making task. Citalopram increased or decreased willingness to exert effort for reward, depending on whether baseline motivation was high or low, respectively. A task assessing decision-making under risk revealed higher levels of risk aversion for potential losses in PD patients, which neither serotonin nor the patient’s regular dopaminergic medication seemed to restore. However, citalopram in PD was associated with more risk-seeking choices for gains, although patients and controls did not differ on this at baseline.

**Conclusion:**

The results provide evidence for a role of the serotonergic system in influencing some aspects of motivated decision-making in PD processes.

## Introduction

Impairments of decision-making and neuropsychiatric symptoms in Parkinson’s disease (PD) have been traditionally studied in the context of dopaminergic dysfunction (Adam et al., 2013; Chong and Husain, 2016; Chong et al., 2015; McGuigan et al., 2019; Moonen et al., 2017; Pagonabarraga et al., 2015). However, there is now increasing evidence that dopamine loss might not account for all such changes and that serotonin too plays an important role. Serotonergic neurons in the median raphe nuclei appear to be affected *early* in the progression of PD-related Lewy body pathology and Lewy neurite deposition, potentially even before dopaminergic cells in the midbrain (Braak et al., 2004; Politis et al., 2010). Several investigations have also been able to directly link this serotonergic dysfunction to neuropsychiatric symptoms, such as apathy or depression. For example, a small investigation using Positron Emission Tomography (PET) imaging with dopaminergic and serotonergic presynaptic transporter radioligands revealed specific serotonergic degeneration in the basal ganglia in PD patients with apathy, compared to those without apathy (Maillet et al., 2016). In contrast, both patient groups showed dopaminergic degeneration, which was not correlated with apathy. Further, apathy in PD seems to be responsive to dopaminergic medication only in some, but not all cases (Chung et al., 2016; Muhammed and Husain, 2016; Seppi et al., 2019), suggesting a role for other neurotransmitters.

However, research so far has mainly focused on establishing correlational links between serotonergic function and questionnaire scores for – or diagnoses of – neuropsychiatric symptoms in PD. What has not been extensively investigated to date is how the modulation of serotonin in PD might affect operationalised, behavioural decision-making or emotion processing measures. Doing so might allow dissection of complex constructs such as motivation or mood deficits into their underlying neurocognitive components, each of which might potentially be differentially related to serotonergic function.

For example, apathy – a loss of motivation – has been operationalised as a reduced goal-directed behaviour that may be caused by reduced sensitivity to the reward that an action may be associated with, and/or by increased sensitivity to the effort that needs to be exerted to obtain a reward (Le Heron et al., 2017; Pagonabarraga et al., 2015). Previously, these decision-making concepts have been studied extensively in relation to the mesolimbic dopamine system, since dopamine is well known for its role in signalling the magnitude and probability of rewards (Alikaya et al., 2018). However, while dopaminergic neurons have been thought for long as the main encoders for reward signals, it has become clear that serotonergic neurons, too, are activated by both expected and unexpected rewards, during their anticipatory and consummatory phases (Li et al., 2016). In addition, a recent probe into effort- and reward-based decision-making in apathetic PD patients highlighted distinct effects of apathy and dopamine on behaviour: While apathy was associated with reduced incentivisation by low rewards, dopaminergic medication increased responding to high reward, high effort options (Le Heron et al., 2018). Thus, dopamine could not reverse the behavioural pattern related to apathy, indicating the importance of exploring the role of alternative neuromodulators which might have implications for treatment options.

Further, PD has been associated with several decision-making and emotion processing impairments, such as risk-seeking, reduced reward and punishment learning or negative bias in information processing (Harmer et al., 2009; Porter et al., 2010). Importantly, all these processes have been linked to serotonergic function. For example, a negative affective processing bias in facial emotion recognition often found in Major Depressive Disorder seems to be responsive to serotonergic treatment. Performance improved in a sample of depressed participants after just 2 weeks of treatment with the selective serotonin reuptake inhibitor (SSRI) citalopram, which correlated with clinical outcome after 6 weeks (Tranter et al., 2009). The authors hypothesised that SSRIs might improve the mood secondary to a positive bias in emotion processing. Thus, if serotonergic function is decreased in PD, it may over time lead to disruption of mood, manifesting ultimately in depression.

Taken together, these studies suggest that researching serotonergic function in PD in more detail might potentially be useful for improving current understanding of these psychiatric symptoms. The aim here was therefore to investigate how serotonergic modulation through administration of citalopram (20 mg) affects decision-making and emotion processing in patients with PD using an experimental medicine approach. Citalopram is an SSRI that blocks reuptake of serotonin in presynaptic cells through the serotonin transporter, thereby increasing the level of serotonin within the synaptic cleft. It is most commonly used as a drug for depression, but also has anxiolytic effects (Izumi et al., 2006). While clinical trials testing the effects of drugs in the treatment of depression usually involve periods of 6–24 weeks, previous research has validated the usefulness of short-term administration of citalopram for probing the effects of acute serotonergic modulation in the brain. For example, investigations in healthy participants and patients with PD have demonstrated that short-term citalopram can alter emotional and cognitive processing in the absence of any antidepressant response (Harmer et al., 2004, 2009; Scholl et al., 2017; Ye et al., 2014).

The current study was designed to probe serotonergic mechanisms underlying decision-making in PD by measuring behaviour before and after 7 days on citalopram. It was specifically not intended to assess antidepressant effects of the drug which are well established. After a screening visit that involved questionnaires and a medical examination, 20 PD patients received a week-long treatment with either placebo or 20 mg citalopram in a within-subject, cross-over, double-blind design. On day 7 of each drug administration phase, patients were examined on experimental tasks that assess various aspects of motivated decision-making: effort and reward sensitivity, risk aversion as well as a set of questionnaires that index neuropsychiatric symptoms. Further tasks on reward and punishment learning and facial emotion recognition are additionally discussed in the Supplemental Material. Twenty healthy controls of similar age were also tested once on these tasks and questionnaires, without medication.

## Methods

### Study design

This was an experimental medicine study using a double-blind, placebo-controlled within-groups design. We recruited 20 patients with a diagnosis of idiopathic PD made according to Parkinson’s UK Biobank criteria via an outpatient clinic (Hughes et al., 1992). Patients were approached and informed about the research study during their regular attendance at the clinic or – if they previously agreed to be contacted for research – via phone. Those with a diagnosis of familial or atypical PD as well as those on current treatment with Monoamine Oxidase B (MAO-B) inhibitors (contraindicated with SSRIs) were excluded. In addition, 20 age-matched healthy controls were recruited from the general population. Ethical approval was provided by the Oxford Research Ethics Committee B and all participants gave written informed consent. Further inclusion criteria for both patients and healthy controls were: aged between 50 and 85 years; no history of or current diagnosis of Diagnostic and Statistical Manual of Mental Disorders, Fifth Edition (DSM-V) bipolar disorder, schizophrenia or eating disorders; no history of or current use of medication for psychosis or depression (including SSRIs) and no history of or current stimulant abuse.

**Figure 1.**
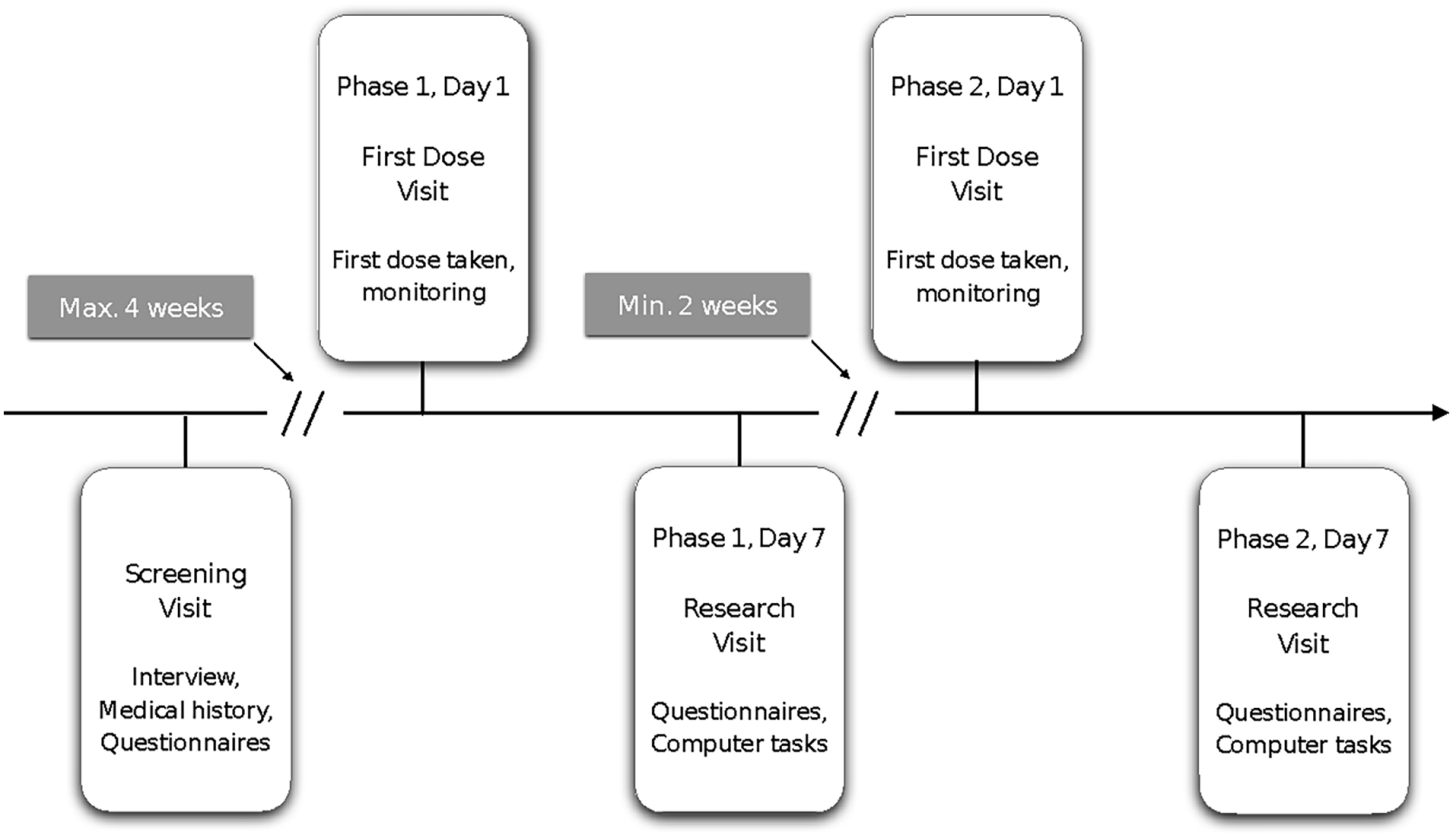
Schematic outline of study design. Patients were invited for a screening visit, and if enrolled, returned for a first dose visit and a research visit for the placebo and citalopram phase each. Placebo and citalopram orders were counterbalanced. Healthy controls were invited for a combined screening and research visit.

Each PD patient received 7 days of citalopram and placebo in a counterbalanced randomised order. The citalopram and placebo tablets were encapsulated to ensure that the two compounds were visually identical. There was a wash-out period of at least 2 weeks between the two phases. With an initial screening visit, two first dose visits (one for drug, the other for placebo) and two research visits (following a week of being on drug or on placebo), the study consisted of five visits for each patient (Figure 1). Controls were tested once without medication.

During the screening visit, patients underwent a medical history review, cognitive assessment and a physical examination to ensure that they were safe to take the citalopram tablets. In addition, they were asked to fill out a set of questionnaires (described below) at screening, and at both research visits. Questionnaire answers at screening were taken as baseline measures for the PD group.

At the first dose visit, patients were pseudo-randomised to receive 7 days of citalopram and placebo in a counterbalanced order. As the sample was stratified by gender at a 1:1 ratio, and the randomisation scheme was performed within each stratum, the allocation is ‘pseudo-random’ as opposed to truly random. This was to ensure that there was a balanced distribution of gender between the two drug administration orders. Although data collection was cut short, this balanced distribution was still met in the current sample. Patients were given the first dose of their assigned compound and were monitored for 1 h after receiving the first dose. At the end of this visit, they received the remaining drug/placebo tablets to take home, with full instructions of when and how to take them.

Testing was completed during the research visit on the morning of day 7 after the first drug/placebo administration. Participants were asked to complete a number of computer-based tasks and fill out the set of questionnaires. Healthy controls completed this research visit once. After a minimum of 2 weeks without any drug/placebo administration, the procedure was repeated beginning from the first dose visit, where patients started the second phase of the study. Since the PD patients were on various PD medication regimens (e.g. once daily vs four times daily) and willingness to withhold medication for study purposes is generally low and biased towards those patients with less severe symptoms, we decided to advise PD patients to continue taking their PD medication as usual during the entire study period.

## Assessments

### Questionnaires

All questionnaires were self-evaluating questionnaires. These included the Hamilton Anxiety and Depression Scale, the Beck Depression Inventory (BDI), the Anxiety Motivation Index (AMI) (Ang et al., 2017), the Unified Parkinson’s Disease Rating Scale, the Geriatric Depression Scale, the State Trait Anxiety Inventory, the Snaith–Hamilton Pleasure Scale, the Toronto Alexithymia Scale (TAS-20) and the Questionnaire of Cognitive and Affective Empathy.

### Effort-based decision-making for rewards

**Figure 2.**
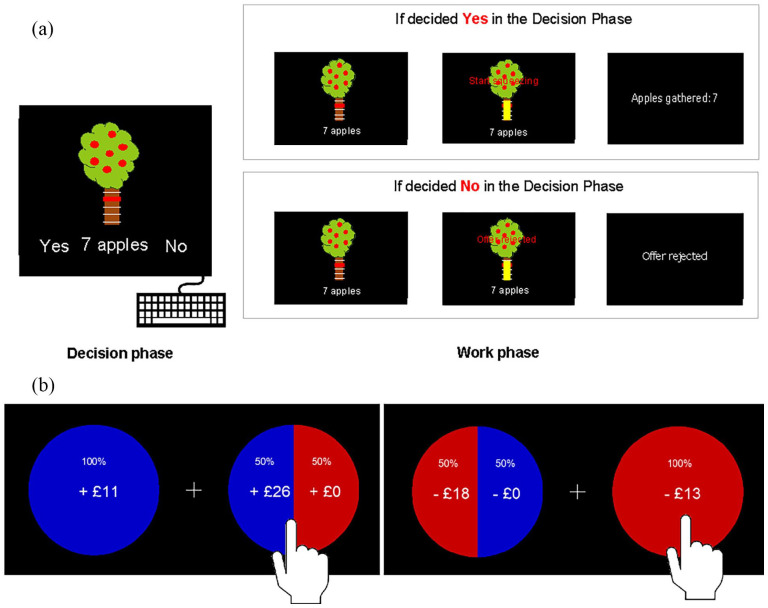
Effort-based and risky decision-making task designs. (a) Effort-based decision-making: The task was divided into a decision and a work phase. In the decision phase, participants were asked to give yes or no responses to offers with different reward and effort levels on a keyboard. Out of 125 decisions in this phase, 10 decisions had to be acted upon in the following work phase, depending on whether the offer was accepted or rejected. Everyone received the same 10 trials to follow through during the decision phase. If an offer was accepted, squeezing the bar to the associated effort level resulted in a gain of apples that translated into money. If an offer was rejected, no action was required. (b) Risky decision-making: This binary choice task represented a risky gamble to increase a win or avoid a loss of money. In the Gain condition, the risky option could result in a larger monetary win than the certain option or result in no win (left). In the Loss condition, the risky option could prevent participants from losing money or make them lose more money than the certain option (right).

This task was designed to estimate reward and effort-based decision-making processes and is explained in detail in Le Heron et al. (2018). Participants were presented with a picture of an apple tree and were asked to react to offers of a certain number of apples (indicative of money) in return for a certain amount of physical effort. Effort was indicated by a red bar positioned on different levels of height on the tree trunk (Figure 2(a)). The main task was to decide whether the effort would be worth the reward. Effort was exerted by squeezing a handle, where the different effort levels were calibrated to a percentage of each individual maximum voluntary contraction (MVC) force. There were five levels of reward (1, 4, 7, 10 and 13 apples), and five levels of effort (16%, 32%, 48%, 64% and 80% of MVC), resulting in a decision space of 25 possible combinations of reward and effort.

The experiment was divided into three parts. First, participants were asked to squeeze the handlebars twice, as hard as possible, to obtain a measure of their MVC force. They were then trained on the different effort levels that were adjusted to their strength. Second, 125 different offers were presented, and for each offer, a *Yes* (accept) or *No* (decline) response had to be made on a keyboard. The positions of the *Yes* and *No* labels on the left and right side of the screen were randomised to prevent biased responses. Before these decision trials, we informed participants that we would randomly select 10 of their decisions, on which they will have to follow through in the end. This was done to ensure that participants would take each trial seriously while minimising fatigue that would occur if they had to squeeze after every trial. In reality, however, the same 10 trials were preselected for everyone: 13 apples for 16% MVC, 7 apples for 48% MVC, 4 apples for 64% MVC, 10 apples for 32% MVC, 10 apples for 16% MVC, 4 apples for 16% MVC, 1 apple for 16% MVC, 7 apples for 32% MVC, 4 apples for 64% MVC and 10 apples for 80% MVC. Thus, in the third part, the handle needed to be squeezed if the offer had been accepted, to obtain the apples and win money (Figure 2(a)). Selecting the same 10 trials allows for a group analysis of force data. However, in this analysis, we only considered yes/no decision data. While the instructions explained that more apples collected translated to more money earned, everyone received the same payment and was debriefed on conclusion of their study involvement.

### Risky decision-making task

In this task, participants were presented with two options to choose from. One option represented a 100% chance of winning a certain amount of money (the certain option), while the other option represented a 50/50 gamble of either winning an amount larger than the certain option, or not winning anything (the risky option, Figure 2(b), left). The position of certain and risky options on the screen was randomised. The task was to consider whether the additional money would be worth taking the risk of not winning any money at all on each individual trial. A response was made by tapping on preferred option. After their response, a new offer was presented, without showing the outcome of the previous choice, or collecting any money that was won. Thus, each offer was to be considered independently of previous choices. A fully risk-neutral decision-maker would choose the risky option whenever its expected value was as high as, or higher than, that of the certain option. For example, if one could be certain to win £5, or take a gamble with 50% probability to win either £10 or nothing, the expected value for each offer would be £5 (Expected value = Probability_Win × Value_Win − (1 − Probability_Win) × Value_NoWin; e.g. Expected value = 0.5 × 10 − (1 − 0.5) × 0 = 5). If one were entirely risk neutral, one would choose the gamble. However, humans are typically risk averse, meaning that the potential win of £10 does not seem worth taking the risk of not winning anything, a well-established phenomenon from economic prospect theory (Kahneman and Tversky, 1979).

In half of the 210 trials, the risky gamble was to avoid losing, rather than to win. In this Loss condition, the certain option represented a 100% chance of losing a certain amount of money, while the risky option involved a 50/50 gamble of either not losing anything or losing more than in the certain option (Figure 2(b), right). For example, consider a trial where the participant has to decide between a certain loss of £12 and a 50% chance of losing £14 or not losing anything (the risky option). Here the expected values are −12 and −7.

There was no time limit, and the offers remained on the screen until a response was given.

### Facial emotion recognition and reversal learning

Supplementary tasks on facial emotion recognition (assessing affective bias) and reversal learning (assessing reward and punishment learning) are discussed in the Supplemental Material.

## Analysis

Tests for differences in demographic variables were done with paired *t*-tests between PD on placebo and on citalopram, and with unpaired *t*-tests between PD on placebo and healthy controls. Questionnaire scores, such as the AMI, were taken from baseline questionnaires collected at the screening visit for PD patients and at the research day for healthy control.

### Effort-based decision-making for rewards

Data from this task were analysed in *MATLAB R*2020*b* (MATLAB, 2020) using custom scripts. Before further analysis, trials with decision times of less than 0.4 s were considered accidental squeezes and therefore removed. Given the hierarchical design of the task, we fit a generalised linear mixed effects (GLME) model with a logistic link function (*fitglme* in *MATLAB*) with binary accept/reject choices for each trial to test for medication effects within the PD patient data only. The full model, including all interactions between reward, effort, apathy and medication status, as well as random intercept and slopes, was compared with all other possible models using the Akaike Information Criterion (AIC) and the Bayesian Information Criterion (BIC). This was done to allow the assessment of all variables of interest and their possible interactions, while also considering the risk of overfitting. The effort term was squared in line with previous literature on perceived effort costs (Morel et al., 2017). All predictor variables were *z*-scored. No model improved fit by >2 AIC or BIC units (chosen as standard cut-off level (Burnham and Anderson, 2004)) over the full model. The winning model included fixed effects for reward, effort, apathy and medication status, a random intercept, and random slopes for reward and effort. The same approach was taken using a depression term instead of apathy, where the depression term was not significant. Because depression scores most likely reflected levels of apathy in the PD group (Supplemental Figure 1), only results covering apathy are reported.

To test for potential session order effects (placebo–citalopram vs citalopram–placebo), the same model was evaluated, this time including a session main effect and Session × Medication interaction term. As the Session × Medication interaction term was not significant, no other coefficient estimations were affected, and model fit did not improve, session was not included in the final model.

In addition, to further investigate the mechanism of potential medication effects, a reduced model including only reward and effort as fixed effects, a random intercept, and random slopes for reward and effort was run. This produced estimations for each participant’s intrinsic motivation (random intercept per person), reward sensitivity (random slope for reward) and effort sensitivity (random slope for effort). We then tested whether these interacted with the level of apathy and medication status with a robust regression using difference scores for each coefficient as the dependent variable, and AMI scores as covariate. Robust regression is an alternative to least squares regression in the presence of outliers, using an iterative re-weighted least squares approach (Yu et al., 2014).

Finally, to test for mean differences in accepted offers between patients on placebo and healthy controls, we ran a one-way ANOVA for the two groups using arcsine transformed choice data. Correlational analyses involving questionnaire (sub-)scores for depression and apathy were done using robust regression because of one outlying score (>2 times standard deviation).

### Risky decision-making task

Two PD patients had incomplete data on this task and were excluded. Binary choice data were analysed on a trial-by-trial basis with a GLME model, using *fitglme* in *MATLAB R*2020*b* (MATLAB, 2020). This was done separately for the Gain and Loss condition. The same procedure as for effort-based decision-making task data was followed here, starting with a full model including fixed effects for the absolute difference in expected value between the risky and certain option in each trial, and for medication, apathy and depression. In addition, the full model included a random intercept, and a random slope for difference in expected value. A term for depression was added here as depression was considered likely to be related to risk taking. All predictor variables were *z*-scored. The winning model was chosen based on AIC and BIC scores, with AIC favouring the full model over any other combination of terms by at least 30 units, and BIC favouring a model with random effects for subject and difference in expected value, a fixed effect for medication, but no terms for apathy and depression, by at least 14 units. Given the small sample size and the large increase in coefficients when using the whole model preferred by AIC, the more conservative winning model indicated by BIC was chosen. Finally, to test for session effects, the same model was fit including a main effect for session (*z*-scored) and an interaction term for Session × Medication.

For both Gain and Loss conditions, the model including session did not improve the fit based on BIC change. Based on AIC change, a model including all main effects and interactions for depression, apathy and session terms had the best fit for loss data (AIC difference = 8.6). However, as this model includes 32 fixed effects coefficients and multiple four-way interactions, we chose to select the model according to the more conservative BIC. The same two models for gains and losses were fit again using only data from PD patients during the placebo phase, and data from healthy controls to test for group effects.

Correlation coefficients were calculated to test for relationships between choice behaviour and measures of apathy and depression with multiple testing corrections applied.

## Results

### Demographics

At baseline, PD patients did not differ significantly from healthy controls in terms of age, gender ratio or cognitive status (Addenbrooke’s Cognitive Examination) (Table 1). None of the participants had dementia or were clinically depressed. Individuals with PD scored slightly higher than controls on a measure of apathy (Apathy Motivation Index (AMI; Ang et al., 2017, 2018; Klar et al., 2022)), depression (BDI (Beck et al., 1961)) and alexithymia (TAS (Bagby et al., 1994)), with differences significant only at the uncorrected level of *α* = 0.05. Importantly, there was no change in any of these questionnaire scores after administration of citalopram.

**Table 1.** Demographics and differences between healthy controls and PD at baseline, and between medication phases.

Measure	Healthy control mean ± SD	PD baseline mean ± SD	Control vs PD baseline *p*-value	PD placebo mean ± SD	PD citalopram mean ± SD	Placebo vs citalopram *p*-value
*N*	20	20	n/a	20	20	n/a
Age	66.95 ± 8.13	68.05 ± 8.40	0.68	See baseline	See baseline	n/a
Gender (F/M)	11/09	10/10	0.75	See baseline	See baseline	n/a
ACE	97.65 ± 2.50	95.45 ± 4.42	0.08	n/a	n/a	n/a
AMI total	0.98 ± 0.42	1.32 ±0.53	0.03*	137 ± 136	136 ±136	0.15
AMI behavioural	0.89 ± 0.56	121 ± 0.62	0.1	133 ± 0.63	122 ± 0.64	0.20
AMI social	118 ± 0.68	161 ± 0.76	0.07	153 ± 0.79	148 ± 0.74	0.46
AMI emotional	0.87 ± 0.46	115 ± 0.50	0.08	124 ± 0.49	119 ± 0.51	0.45
BDI	3.80 ± 4.44	8.55 ± 4.22	0.001**	8.95 ± 6.00	7.85 ± 4.63	0.15
GDS	0.85 ± 2.06	1.85 ± 1.35	0.08	1.74 ± 1.45	1.32 ± 1.16	0.06
STAI – state	22.45 ± 13.25	28.40 ± 7.14	0.09	29.05 ± 7.72	29.50 ± 9.43	0.74
STAI – trait	23.45 ± 13.50	30.70 ± 8.12	0.05	32.30 ± 6.76	31.95 ± 7.74	0.77
SHAPS	0	0.15 ± 0.49	n/a	0.30 ± 0.73	0.25 ± 0.55	0.72
TAS	36.05 ± 5.99	42.10 ± 11.30	0.04*	45.70 ± 1153	44.35 ± 12.01	0.27
QCAE	9140 ± 9.81	92.20 ± 11.57	0.82	95.30 ± 10.97	95.70 ± 1166	0.81
UPDRS	n/a	29.95 ± 2.88	n/a	29.95 ± 15.71	33.16 ± 9.58	0.37

ACE: Addenbrooke’s Cognitive Examination; AMI: Apathy Motivation Index; BDI: Beck Depression Inventory; GDS: Geriatric Depression Scale; QCAE: Questionnaire of Cognitive and Affective Empathy; SHAPS: Snaith–Hamilton Pleasure Scale; STAI: State Trait Anxiety Inventory; TAS: Toronto Alexithymia Scale; UPDRS: Total Unified Parkinson’s Disease Rating Scale Score.

* Significant at *p* < 0.05. **Significant at *p* < 0.01. *p*-Values uncorrected for multiple testing.

Since PD patients differed from controls in total BDI scores, but the BDI included several symptoms that overlap with symptoms of PD itself or apathy, we plotted the distribution of individual symptoms within the BDI questionnaire (Supplemental Figure 1). In both the age-matched control and PD groups, lack of energy and loss of libido were commonly reported. PD patients additionally reported significantly more changes in sleep (*t*(38) = −4.10, *p* < 0.001), fatigue (*t*(38) = −4.13, *p* < 0.001) and concentration problems (*t*(38) = −3.79, *p* < 0.001). Levels of core symptoms of depression, such as sadness, feeling punished, guilty, or like a failure, and suicidality were low in both groups. None of the participants had clinical depression.

### Effort-based decision-making for rewards

In this effort-based decision-making task (Bonnelle et al., 2015), which has been previously used in PD (Chong et al., 2015), participants were asked to accept or reject offers of different amounts of monetary reward (five levels of reward) in return for exerting physical effort on a handheld dynamometer at various difficulty levels (five levels of effort). The AMI was included to measure apathy.

#### PD reduces overall offer acceptance for effortful tasks

**Figure 3.**
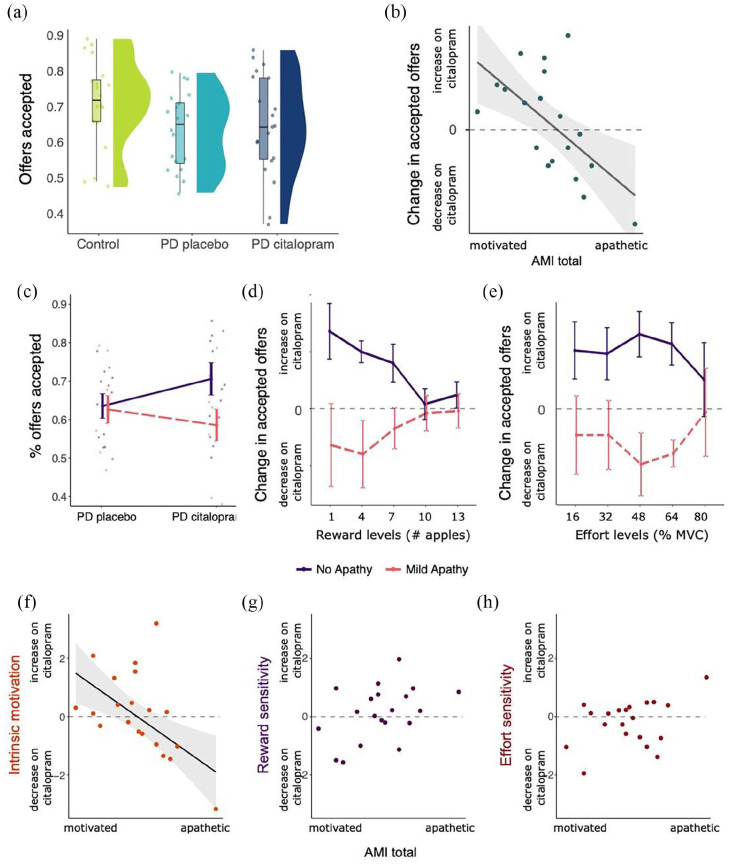
Effects of PD and citalopram on decision-making. (a) Significant difference in the proportion of offers accepted between healthy controls and PD on placebo. There were no mean differences between PD on placebo and citalopram. (b) Significant correlation between change in accepted offers (citalopram–placebo) and AMI scores, reflecting a Medication × Apathy interaction. (c) Visualisation of Apathy × Medication interaction by binary mean split of apathy groups. Citalopram increased the proportion of accepted offers for patients who scored low on apathy, but not for patients scoring high on apathy at baseline. (d) Change in accepted offers (citalopram–placebo) by reward levels, illustrating strongest Citalopram × Apathy interaction for low rewards. This may suggest that citalopram affected processing or sensitivity of low rewards specifically, but in opposing directions for apathetic and non-apathetic patients. (e) Change in accepted offers (citalopram–placebo) by effort levels, showing strongest Citalopram × Apathy interaction for medium effort levels. (f) Intrinsic motivation, calculated with each patient’s random intercept during the citalopram and placebo phases, correlated negatively with level of apathy. Higher levels of apathy were associated with a decrease in intrinsic motivation on citalopram, and vice versa. (g) No significant correlation between change in reward sensitivity and AMI scores. (h) No significant correlation between change in effort sensitivity and AMI scores. Error bars and shaded area represent standard error.

A one-way ANOVA for mean differences in accepted offers between patients with PD and healthy controls showed that overall, patients accepted significantly fewer offers than controls (Figure 3(a); *F*(1, 38) = 4.40, *p* = 0.04). Robust regression between offers accepted and AMI scores for healthy controls and PD on placebo (treated as one group) revealed no relationship (*p* = 0.14). This was also the case for the association between offers accepted and AMI sub-scores for social, emotional and behavioural apathy (all *p* > 0.05). Similarly, none of the other questionnaire scores were significantly associated with offers accepted (all *p* > 0.05).

#### Citalopram interacts with baseline motivation

Binary choice data (accept/reject) were analysed using a hierarchical GLME model. Change scores for the proportion of accepted offers (citalopram–placebo) were correlated with apathy severity, indexed by the AMI questionnaire. The selected GLME model included significant two-way interactions for Reward × Effort, Effort × Apathy and Medication × Apathy. In addition, there were significant three-way interactions for Reward × Effort × Apathy and Reward × Apathy × Medication (see Supplemental Table 1 for fixed effects coefficients and significance).

The variables that had the strongest impact on whether offers were accepted or rejected were the associated effort and reward, as indicated by the size of their regression coefficients. Participants were more likely to except an offer with larger reward (positive Reward coefficient, *p* < 0.001), and less likely to accept one with higher effort (negative Effort coefficient, *p* < 0.001). In addition, higher effort levels affected the acceptance of lower reward offers more strongly than the acceptance of higher reward offers (Reward × Effort interaction, *p* < 0.001), reflecting that both effort and reward processing were involved in the decisions in this task.

Importantly, the effect of citalopram on offer acceptance depended on patient’s levels of apathy (Apathy × Medication interaction, *p* = 0.001). This can be visualised by plotting the difference in accepted offers between citalopram and placebo phases against total AMI scores (Figure 3(b)). Higher apathy scores were associated with a decrease in accepted offers on citalopram, while lower apathy scores were related to increased acceptance on citalopram (*r* = −0.62, *p* = 0.003). Thus, more motivated patients were more willing to perform effort for reward on citalopram, while more apathetic patients were less willing to do this on the drug. Regarding subscales of the AMI, there was a very strong correlation between the change in accepted offers on citalopram and the social subscale of the AMI (*r* = −0.68, *p* < 0.001). The correlation with the behavioural subscale was significant at the uncorrected alpha level (*α* = 0.05, *r* = −0.46, *p* = 0.04), but did not remain significant after correcting for multiple testing (*α* = 0.01). The emotional subscale was not related to change in accepted offers (*p* = 0.09), nor were any of the other questionnaires (all *p* > 0.05).

By classifying patients into non-apathetic (*n* patients = 10) and mildly apathetic (*n* patients = 10) groups based on a mean split, it is apparent that apathy was not associated with differences in accepted offers in the placebo phase. As shown in Figure 3(c), the mean percent offers accepted are not different between the two groups in the placebo phase. The effect of citalopram in relation to apathy can be further investigated by plotting the change in accepted offers across the different reward (Figure 3(d)) and effort (Figure 3(e)) levels. Citalopram increased accepted offers in patients without apathy especially for low reward. In contrast, the error bars for the effect of citalopram in patients with and without apathy overlapped for the highest effort level, but not for the other effort levels. This may suggest that citalopram affected processing or sensitivity of low rewards and medium effort levels specifically, but in opposing directions for apathetic and non-apathetic patients.

Finally, we estimated each patient’s intrinsic motivation (random intercept per person in GLME), reward sensitivity and effort sensitivity to examine which aspects of motivated behaviour might be affected by apathy and citalopram. Figure 3(f) to (h) shows significant relationships between change scores (citalopram–placebo) and total AMI scores for intrinsic motivation, calculated with robust regression because of an outlier (f; *b* = −0.27, *p* = 0.01). This was not the case for reward (g; *b* = 0.39, *p* = 0.05) or effort sensitivity (h; *b* = 0.36, *p* = 0.15). AMI sub-score analysis revealed a significant association between the social subscale and intrinsic motivation (*b* = −0.41, *p* = 0.004), but not other subscales. Similarly, no subscale associations for reward or effort sensitivity reached significance. Thus, in patients with higher apathy scores, citalopram was associated with a *decrease* in intrinsic motivation, while lower apathy scores were associated with an *increase* in intrinsic motivation on citalopram.

### Reward- and punishment-based decision-making under risk

**Figure 4.**
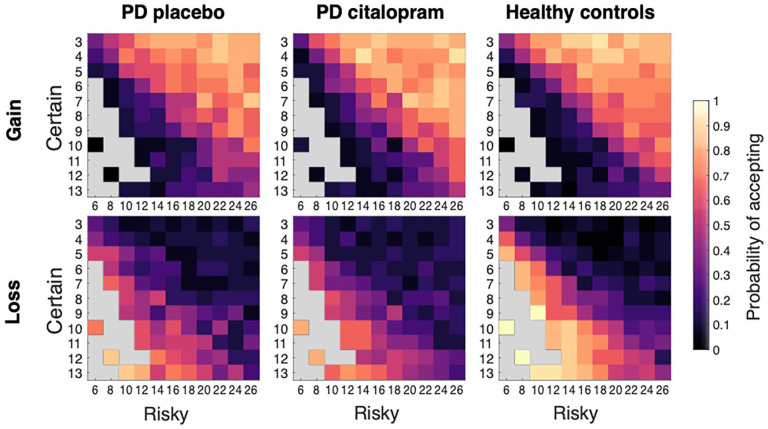
Choice heatmaps for risky decisions. Numbers on *X*- and *Y*-axes represent monetary values for risky and certain options, respectively. Grey squares represent missing combinations of values, where the certain option is larger or equal to the risky option, except for some catch trials. In the Gain condition, participants were more likely to choose the risky option when its expected value (= money to win) was larger than the certain offer. In the Loss condition, they were more likely to choose the risky option when the expected value (= money to lose) was lower than the certain offer.

In a different task, participants were asked to choose between two offers: A certain win of money, or a risky – larger – win, with a 50% chance of not winning anything. The offers could also involve a certain loss and a 50/50 gamble to lose more or not lose anything. Binary choice data were analysed with a GLME model and are visualised best on a heatmap depicting likelihood of accepting offers as a function of risk and certainty for gains or losses (Figure 4).

#### Citalopram decreased patients’ risk aversion for gains

The GLME model for data from the Gain condition included two significant fixed effects, difference in expected value and medication. As anticipated, participants were more likely to choose the risky option (>50% of choices), when its expected value was higher than the certain option (*b* = 2.22, *p* < 0.001; Figure 4 top panel shows the probability of accepting an offer in the Gain condition for different expected values).

**Figure 5.**
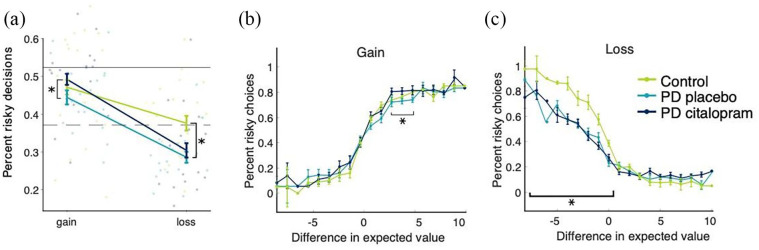
Effect of medication and PD on risky decisions. (a) PD patients on citalopram were less risk averse for gains than during placebo phase. PD patients were generally more risk averse for losses than controls. The upper horizontal line represents the border for risk neutrality in the Gain condition, the dashed horizontal line represents the border for risk neutrality in the Loss condition. (b) Patients on placebo accepted slightly fewer risky options for offers with expected values between 1 and 5 (i.e. difficult decisions) than patients on citalopram. (c) Healthy controls accepted more risky offers whenever the expected loss was lower than in the certain offer, reflecting more rational decision-making than patients on placebo. Error bars represent standard error.

In addition, patients were more likely to choose the risky option when on citalopram, compared to placebo (*b* = 0.12, *p* = 0.01, Figure 5(a)). This effect also depended on the difference in expected value between risky and certain offers (interaction *b* = 0.12, *p* = 0.03, Figure 5(b)). On citalopram, patients chose the risky option more often, especially for offers where the difference in expected value (*expected value of certain offer* − *expected value of risky offer*) was between 0 and 5.

While there was no main effect of group when comparing mean choices of patients with PD on placebo and healthy controls (main effect group *b* = 0.006, *p* = 0.90), there was a Group × Expected value difference interaction (*b* = 0.14, *p* < 0.001). Healthy controls had a steeper slope for the increase in risky choices with increasing expected value differences. The overall slope of the increase in risky choices going from negative to positive expected values is slightly steeper for controls than the slope for PD patients on placebo, Figure 5(b)).

#### PD associated with increased risk aversion for losses

In the Loss condition, participants were more likely to choose the risky option when the expected value (here, money to lose) was lower than for the certain option (Figure 4 lower panel, main effect difference: *b* = −1.65, *p* < 0.001). Citalopram did not affect decision-making in the Loss condition (*b* = 0.02, *p* = 0.68, Figure 5(c)), and there was no interaction between citalopram and difference in expected value to lose (*b* = 0.08, *p* = 0.18, Figure 5(c)).

However, in the model including only PD patients on placebo and healthy controls, a significant main effect of group suggests that PD patients chose fewer risky options than controls (main effect group, *b* = 0.14, *p* = 0.005, Figure 5(a)). In addition, the difference in risky choices between PD and controls depended on the expected values between risky and certain offers (interaction effect Group × Difference, *b* = −0.37, *p* < 0.001, Figure 5(c)). PD patients selected fewer risky options only for trials in which the risky offer was objectively preferable for a risk-neutral decision-maker. In the Loss condition, the lower the expected value difference, the lower the expected loss of money. Thus, a rational decision-maker would always choose the risky option if the expected value difference were lower than zero, meaning that PD patients showed more risk aversion than controls. Finally, there were no significant correlations between the medication effect on gain or loss choices and any of the questionnaire scores (all *p* > 0.05).

### Facial emotion recognition and reversal learning

There were no effects of citalopram on facial emotion recognition (assessing affective bias) and reversal learning (assessing reward and punishment learning) within the PD group. More detailed results are discussed in the Supplemental Material.

## Discussion

In this within-subject, placebo-controlled cross-over experimental medicine study, we investigated how a 7-day administration of 20 mg citalopram affected mechanisms of decision-making and emotion processing in PD. Results indicated that PD patients were overall less motivated (Figure 3, Table 1) and more risk averse for potential losses (Figure 5(a) and (c)) than healthy controls but did not differ in terms of reward or punishment learning (Supplemental Figure 2) or facial emotion recognition performance. We further found that citalopram had complex effects on decision-making, depending on baseline levels of motivation.

### Citalopram increased intrinsic motivation only in already motivated patients

At baseline, PD patients scored higher on levels of apathy (AMI score), and thus had lower levels of motivation than healthy controls. A novel finding was that the effect of citalopram on motivated behaviour (accepted offers) depended on *baseline levels of motivation* as measured by the AMI. Citalopram increased acceptance of offers only in patients who scored low on the apathy measure, thus were already motivated, while this effect was reversed for patients who were more apathetic (Figure 3). In an already motivated state, citalopram increased, while in an apathetic state it decreased acceptance of offers, especially for low reward and low-to-medium effort offers. In a previous report using this task, Le Heron et al. (2018) tested PD patients with and without diagnosed apathy, while ON and OFF their usual dopaminergic medication. They described that apathy was associated with reduced responding to low reward outcomes while OFF dopaminergic medication, concluding that apathy might be associated with reduced incentivisation by reward specifically. Apathetic patients still agreed to high effort offers, but only if the associated reward was large. In comparison, the current study found that apathy was linked to reduced choices of low reward outcomes during citalopram administration, but not during the placebo phase (i.e. usual dopaminergic medication state).

It has previously been argued that SSRIs might actually exacerbate apathy symptoms in PD patients.^28^ As apathy levels were low overall in the current study, our mildly unmotivated patients may have experienced a further citalopram-induced reduction of motivation to a level that we could detect in task performance. However, the interaction between apathy and citalopram was linked to changes in intrinsic motivation and effort sensitivity, not changes to reward sensitivity. This might point to a slightly different process through which serotonin is involved in reward and effort-based decision-making. More work is required to develop a full picture of this pattern, for example by modulating serotonin in the opposing direction, such as with acute tryptophan depletion (ATD). One previous study reported a small but significant increase in depression scores with ATD, but this occurred in both the PD and healthy control group (Mace et al., 2010).

The interpretation of these findings is complicated by the fact that most of the patients tested in the current study were taking dopaminergic medication, and of these, all were tested while on their usual regimen. It is possible this influenced outcomes; this will need to be further investigated in future cross-over studies. In Le Heron et al. (2018), an effect of dopaminergic medication was primarily observed for acceptance of high effort, high reward offers. Crucially, the authors did not find an interaction between apathy and dopamine, such that the effect of dopamine on offer acceptance rate did not depend on levels of motivation. This may suggest that while dopamine influences goal-directed behaviour especially in terms of reward valuation, serotonin may be more involved with a baseline ‘readiness’ to act or overcome effort.

Intriguingly, the correlation between the change in motivated behaviour on citalopram and baseline motivation was strongest for the social subscale of the AMI, which measures levels of social engagement. A role for serotonin in social motivation is in accord with reports of increased prosocial behaviour after increasing serotonergic levels in the brain, for example by administration of citalopram or 3,4-methylenedioxymethamphetamine (ecstasy) (Crockett et al., 2010; Hysek et al., 2014). Establishing whether social, emotional and behavioural forms of apathy may be differentially influenced by neurotransmitter function may help decode the vast individual differences in response to drug treatment for apathy.

### Citalopram increased rational risk-seeking for gains depending on expected value

On citalopram, PD patients were more likely to choose the risky option when playing for rewards, but not when playing to avoid losing (Figure 5). Choices of risky options for gains increased towards the risk-neutral boundary in PD patients during the citalopram phase. In addition, the effect of serotonin on risk-seeking in our task was strongest for offers where the difference in expected value was small (Figure 5(b)). Thus, patients on citalopram required smaller rewards as incentive to switch their preference to the risky option than on placebo. This difference between medication phases decreased towards the more extreme offers.

One conclusion might be that in the presence of risk, serotonin is involved in the regulation of reward-seeking, but not punishment-avoidant behaviour. This is somewhat surprising, as serotonin is generally thought to be more closely involved in punishment processing than reward processing (Cools et al., 2011; Tanaka et al., 2009). In addition, the 1-week administration of citalopram in this study was associated with increased risk-seeking for gains. This is in contrast to an investigation in primates that increased risk-seeking after *reducing* levels of serotonin through ATD (Long et al., 2009).

### PD associated with decreased rational risk-seeking for losses

In contrast to the Gain condition, we did not find a medication effect in the Loss condition. PD patients on their usual dopaminergic medication were still more risk averse than healthy controls for all offers where rational decision-making was required to overcome risk aversion for loss, that is, offers where the certain option was not necessarily preferable. Citalopram did not reverse this difference. Healthy controls performed remarkably close to a fully rational decision-maker. A previous report using the Vancouver Gambling Task reported a small effect for PD while OFF medication, with increased risk aversion for large losses (Sharp et al., 2013).

Thus, PD seems to be associated with changes in decision-making under risk, though neither serotonin, at least as increased by the 7 days of citalopram given here, nor the patient’s regular dopaminergic medication could restore these changes to normal. If, as often hypothesised, increasing the level of serotonin does increase risk aversion, while dopaminergic medication is thought to increase risk-seeking, these two effects may have cancelled each other out in the Loss condition. While we observed a different direction of the effect of serotonin in the Gain condition, it is possible that serotonin affects reward and punishment processing differently, as argued above (Cools et al., 2011; Kranz et al., 2010; Tanaka et al., 2009).

## Conclusion

This study provides further evidence that PD is associated with motivational deficits and that the serotonergic system influences these processes. In motivated patients (without apathy), citalopram led to *increases* in willingness to act, rather than *decreases*, as often reported in healthy volunteers. Disentangling the functions of serotonin has been notoriously difficult, partly because the serotonergic system likely strongly interacts with other neurotransmitters and neuromodulators, like dopamine (Liu et al., 2014; Qi et al., 2014). While these interactions are highly complex, what stands out is that dopamine and serotonin are interdependent for encoding reward and punishment signals. In the case of PD, precision medicine approaches might require better profiling of each patient’s symptoms to achieve more effective individualised treatment.

## Supplemental Material

sj-docx-1-jop-10.1177_02698811221144636 – Supplemental material for Role of serotonin in modulation of decision-making in Parkinson’s disease Supplemental material, sj-docx-1-jop-10.1177_02698811221144636 for Role of serotonin in modulation of decision-making in Parkinson’s disease by Lisa Nobis, Maria Raquel Maio, Youssuf Saleh, Sanjay Manohar, Annika Kienast, Emily McGann and Masud Husain in Journal of Psychopharmacology
