# Supplementary material for: Role of serotonin in modulation of decision-making in Parkinson’s disease
Source: J Psychopharmacol. 2023 Jan 11;37(4):420–31. doi: 10.1177/02698811221144636 (PMC10101180; doi:10.1177/02698811221144636)
Supplement: sj-docx-1-jop-10.1177_02698811221144636 – Supplemental material for Role of serotonin in modulation of decision-making in Parkinson’s disease [file sj-docx-1-jop-10.1177_02698811221144636.docx]

# Supplementary Results

## BDI mean scores


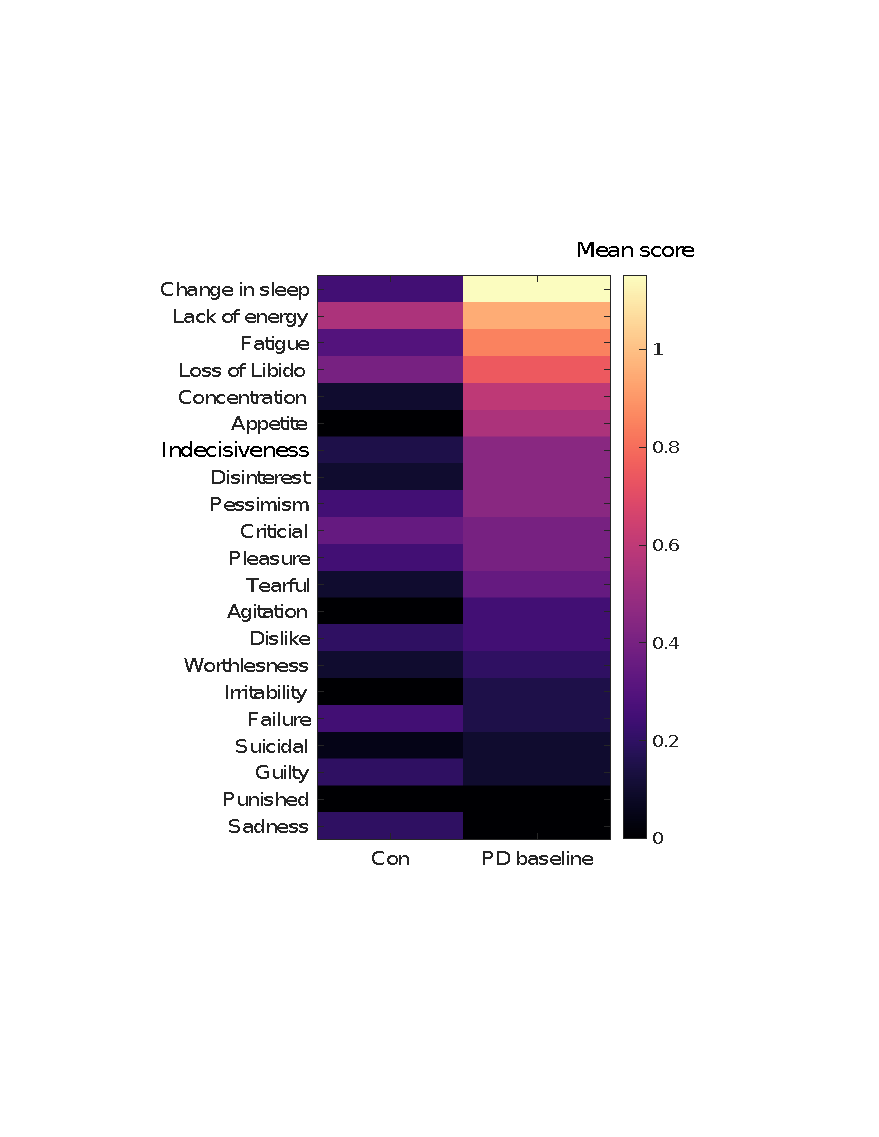


**Figure S2: Mean scores per BDI questionnaire item.** Items are sorted by severity in the PD group. Lighter fields represent more severe symptoms, such that symptoms of sleep problems, lack of energy, fatigue, and loss of libido were most common in PD. Core symptoms of depression, such as sadness, feelings of guilt, or thoughts of suicide were least common in the PD group. In the control group, lack of energy and loss of libido were most commonly reported.

## Effort-based decision-making task


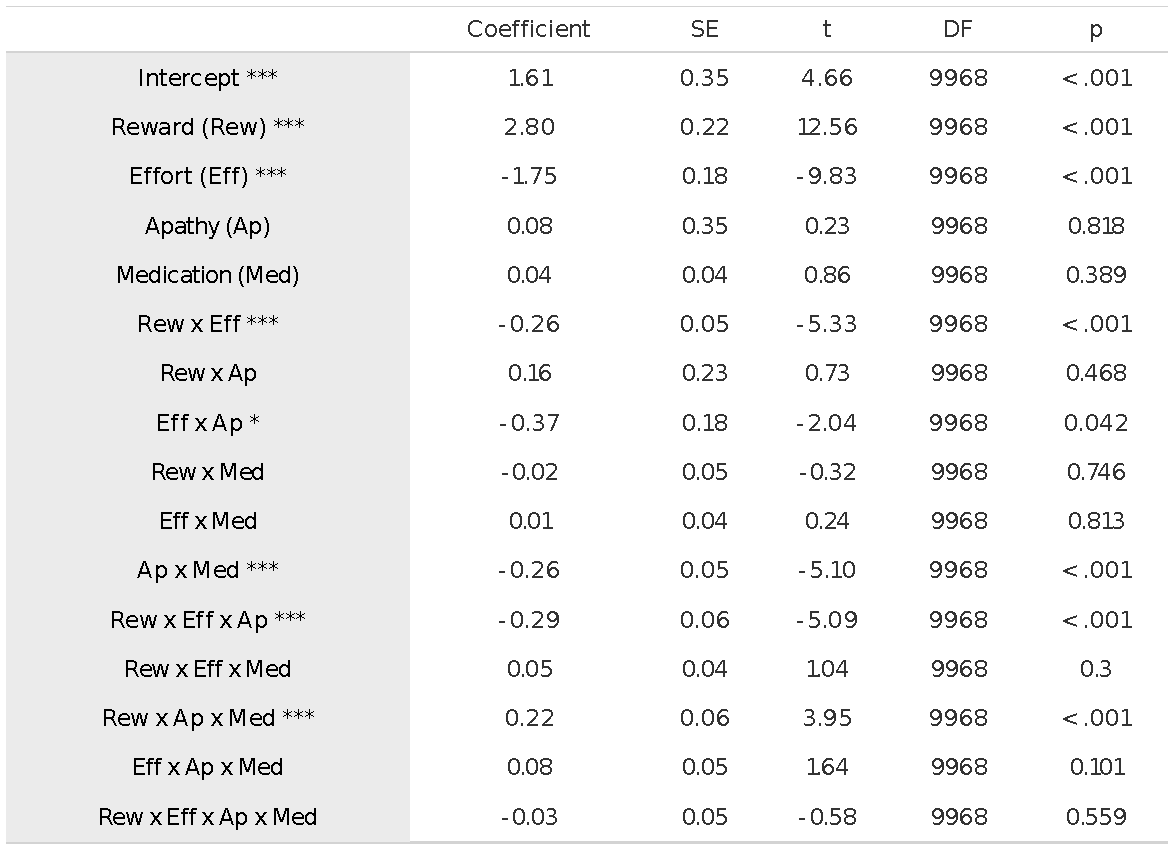


**Table S1: Apples GLME model coefficients.**

Note: * significant at p <.05; ** significant at p <.01; *** significant at p <.001.

## Reversal learning task

To measure potential effects of citalopram on learning of reward and punishment associations, we included a simple reversal learning task, where participants had to learn underlying winning probabilities of two decks of cards by trial and error. After between 16-20 trials, these probabilities reversed. The goal was to maximise winnings.

### No effects of citalopram on reversal learning

There were no significant differences in accuracy (i.e. percentage of high-probability choices) either between the placebo and citalopram phase (paired t-test, *p* > .05), or between PD on placebo and healthy controls (unpaired t-test, *p* > .05). Accuracy improved equally in all groups as the number of trials since reversal increased. A two-way, repeated measures ANOVA with mean choices as dependent variable, and choice type (win-stay vs. lose-switch) as well as medication status as within-subject factors, revealed a significant effect of choice type (*F*(1,18) = 26.44, *p* < .001), but not of medication (*p* = .80). Patients were more likely to stick to an option when the previous trial won, than to switch when the previous trial lost. Similarly, patients on placebo did not differ from healthy controls in their win-stay and lose-switch behaviours, as indicated by a repeated measures ANOVA with group membership (PD placebo vs. healthy control) as between-subjects factor (*p* = .24).

As reaction time data was positively skewed and differed throughout the course of the experiment, medication effects were calculated using a GLME with reaction time as dependent variable, and medication status, reversal block, and session order as predictors. Session order had the strongest impact on reaction time, decreasing reaction time by .07 units (*p* < .001). Patients also responded faster with increasing number of blocks completed (*b* = -.06, *p* < .001). Importantly, citalopram significantly decreased reaction time by .05 units (*p* < .001), but did not interact with reversal block or session order. In other words, citalopram’s effect on reaction time was independent of the learning rate, and not confounded by session order.

A separate GLME was run to test for differences between PD on placebo and healthy controls. The winning model for this design included only a fixed main effect for reversal, a random intercept and a random slope for reversal, but no significant group differences.

There were no significant correlations between accuracy or reaction time and questionnaire scores (*p* > .05).


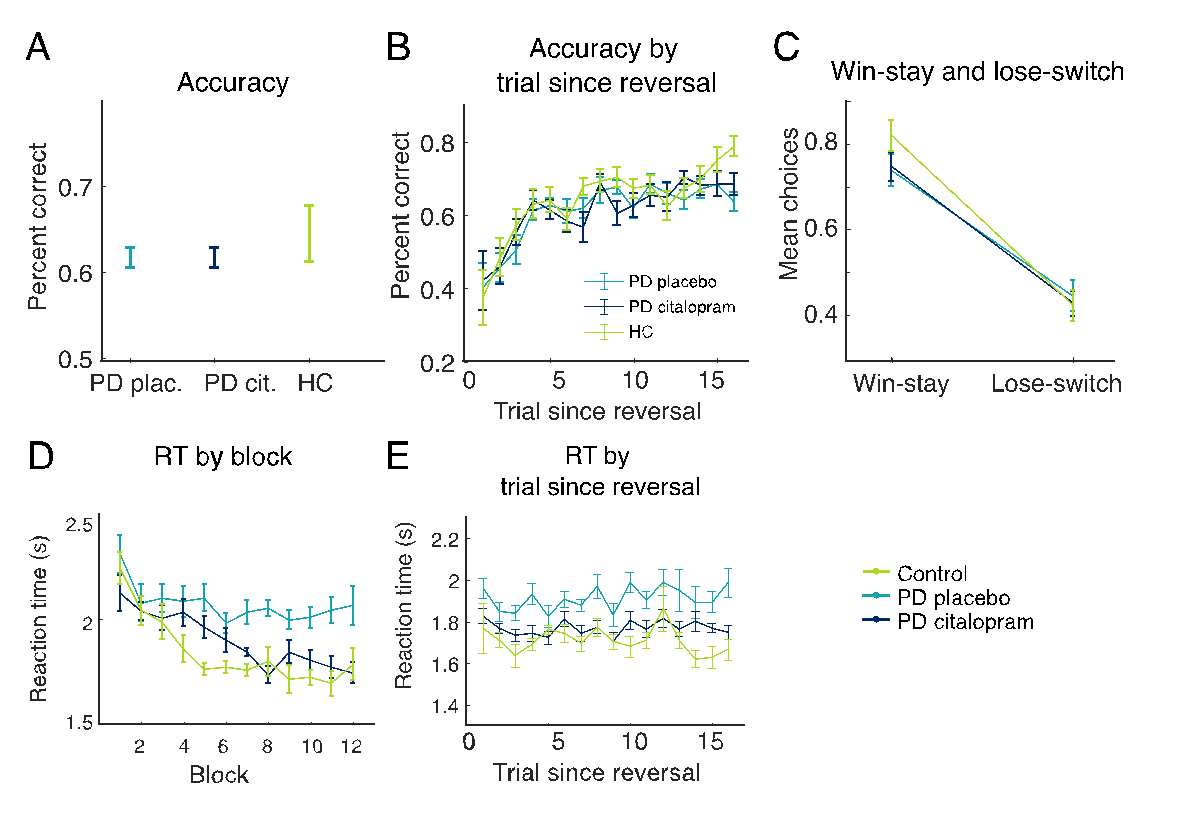


**Figure *S3*: Choice behaviour and reaction time on reversal learning task. A.** No group or medication effects on accuracy, indicated by percent correct (i.e. high-probability) choices. **B**. Accuracy time-locked to trial since reversal, averaged over all blocks, reflecting learning rate. As more trials since reversal are completed, accuracy improves equally for all groups. **C**. All groups were more likely to stay with an option after winning (win-stay), than to switch to another option after losing (lose-switch). **D.** RT from first to 16^th^ trial since reversal, averaged over all blocks, thus indicating average learning rate. PD on placebo were slower to respond independent of learning than PD on citalopram. **E.** RT from first to 12^th^ block of reversals, averaged over all trials within each block, thus indicating task difficulty. PD patients on placebo have larger RT throughout the whole experiment. While there seems to be a trend for larger RT differences towards later blocks, this interaction was not significant. Error bars represent standard error.

This is in contrast with a number of previous studies, as a general impairment in reversal learning especially for learning from negative rather than positive outcomes is commonly found in PD (Cools et al., 2011; Cools and D’Esposito, 2011; Graef et al., 2010; Peterson et al., 2009). One possible explanation for the lack of a difference in this sample is that the relatively few number of trials in each reversal block may have created an especially difficult task for both PD patients and healthy controls. However, **Figure S3 B** supports the assumption that some learning did take place within each block.

During the citalopram phase, patient’s reaction times were significantly shorter relative to the placebo phase. This effect was robust against correcting for practice effect. While there was no significant reaction time difference between patients on placebo and controls, **Figure S3 D** suggests a trend that may have suffered from a lack of power for between-subject testing given the small sample size. Thus, the most likely interpretation is that citalopram corrected a reaction time slowing in PD relative to healthy levels. Although this particular finding has not been reported in PD before, it is in line with one previous investigation of serotonergic modulation in PD. Ye et al. (2014) documented that citalopram administration in PD reduced reaction times in a stop-signal task, an effect that depended on disease severity. The authors concluded that citalopram may have counteracted the progressive loss of forebrain serotonergic projections in patients with more advanced disease (Ye et al., 2014).

Another piece of evidence for the involvement of serotonin in reaction times is an analysis of the effects of ATD in healthy volunteers, thought to *decrease* serotonin levels, which resulted in slowed responding during a visual discrimination and reversal learning task (Murphy et al., 2002). Similar to the findings here, the authors did not find impaired performance following ATD, concluding that the decrease in serotonin levels may have affected inhibitory control, which was compensated for by the participants through increased deliberation times. Following this line of reasoning, the PD patients in the current sample may have overcome reduced inhibitory control by slowing reaction times, a process that was no longer necessary with potentially replenished serotonin levels during the citalopram phase.

## Facial Emotion Recognition task

In this task, participants were presented with a picture of an averaged face showing an emotion to some extent. The picture disappeared after 500ms, and participants had to select the emotion they identified in the stimulus in a forced choice of 6 possible responses. After their response, participants were asked to rate the strength of the shown emotion, and their confidence in their response.

There were no significant within-subject effects for citalopram, or between-subject effects for PD, in accuracy, question ratings, or reaction time (*p* > .05) as analysed with repeated-measures ANOVA. In addition, there were no effects for apathy and depression in accuracy or reaction time (*p* > .05). There was a significant main effect for emotion in both the medication models and the healthy control models, for all four dependent variables (all *p* < .001). Overall, participants were most accurate, most confident, reported highest intensity, and responded fastest for happy faces, while they were least accurate, least confident, reported lowest intensity and responded slower for sad, afraid, and angry faces.

The main effect for medication was not significant (*p* =.08). There was an interaction between apathy and emotion (*F*(5,75) = 3.87, *p* =.004, *n2p* =.2) for ratings on question one (“How sure are you about your response?”). Participants scoring higher on the AMI (*n* = 10) tended to report lower confidence in most expressions, except happy and surprised faces. None of the questionnaire scores correlated significantly with change in accuracy, reaction time, or responses on the two questions (all *p* > .05).

## Emotion recognition control task

This task was added as a control to the facial emotion recognition task, and was intended to measure visual acuity for emotional facial expressions. Participants were presented with a target facial expression, and a neutral expression of the same face. When they moved a sliding scale shown on the screen, the neutral expression slowly changed into the target facial expression (e.g. from neutral up to 100% anger, in increments of 5%). The goal was to find the position on the sliding scale were both images looked as similar as possible to each other.

Participants were most precise for happy faces, and least precise for sad and angry faces (main effect emotion, medication model: *F*(5,75) = 6.96, *p* < .001; healthy control model: *F*(5,175) = 17.96, *p* < .001). There were no significant differences between placebo and citalopram phases, or between PD and healthy control, on precision in facial emotion expression matching (*p* > .05). There were also no effects for apathy or depression, nor were there significant correlations between precision and questionnaire scores (all *p* > .05).

These results contrast with a recent meta-analysis of emotion recognition studies in PD, which concluded that there was enough evidence to support a slight emotion recognition impairment in patients with PD (Argaud et al., 2018). However, these findings have been criticised for being confounded with visual and executive impairments in PD (Argaud et al., 2018; Hipp et al., 2014). For example, it has been argued that facial expressions of negative emotions have less prominent visual discriminatory features than expressions of happy emotions, and are thus more visually challenging to recognise. In the presence of visuospatial or executive function deficits, patients with PD might be especially prone to making errors with negative faces. Since most reports describe a deficit only in these potentially more challenging conditions, the differences may be mainly due to visual factors. This is supported by the fact that there were no differences in *Twins* performance. However, as levels of depressive symptoms were very low in this sample, an open question remains whether changes in emotion processing after citalopram treatment could be observed in PD patients with more severe depression.

Participants who scored high on the AMI tended to report less confidence in their response than those who scored low on the AMI. One explanation may be based on the theory that apathy involves higher levels uncertainty in decision-making. For example, with the AMI we asked patients to rate their agreement on statements like “I make decisions firmly and without hesitation” or “After making a decision, I will wonder if I made the wrong choice”. Higher scores on these questions may reflect general uncertainty. It could also be argued that unmotivated patients exerted less effort into their response, and therefore reported less confidence. However, as there were no differences in accuracy or reaction times between motivated and unmotivated patients, this explanation seems unlikely. If a relationship between apathy and reduced confidence in decisions can be replicated in other tasks, this could have important implications for the development of cognitive-behavioural therapies for apathy.

Cools R and D’Esposito M (2011) Inverted-U–shaped dopamine actions on human working memory and cognitive control. Biol Psychiatry 69: e113–e125.

Graef S, Biele G, Krugel LK, et al. (2010) Differential influence of levodopa on reward-based learning in Parkinson’s disease. Front Hum Neurosci 4: 169.

Murphy FC, Smith KA, Cowen PJ, et al. (2002) The effects of tryptophan depletion on cognitive and affective processing in healthy volunteers. Psychopharmacology 163: 42–53.

Peterson DA, Elliott C, Song DD, et al. (2009) Probabilistic reversal learning is impaired in Parkinson’s disease. Neuroscience 163: 1092–1101.
